# External walking environment differentially affects muscle synergies in children with cerebral palsy and typical development

**DOI:** 10.3389/fnhum.2022.976100

**Published:** 2022-09-23

**Authors:** Yushin Kim, Thomas C. Bulea, Diane L. Damiano

**Affiliations:** ^1^Department of Sports Rehabilitation, Cheongju University, Cheongju, South Korea; ^2^Neurorehabilitation and Biomechanics Research Section, Rehabilitation Medicine Department, National Institutes of Health, Bethesda, MD, United States

**Keywords:** muscle synergy, cerebral palsy, coordination, gait, electromyography

## Abstract

Despite external environmental changes in walking, such as manipulating gait speed, previous studies have shown that the underlying muscle synergy structures (synergy weights or vectors) rarely vary. The purpose of this study is to examine if external environmental changes to the walking task influence muscle synergies in children with cerebral palsy (CP) and/or typical development (TD). To identify muscle synergies, we extracted muscle synergies from eight children with CP and eight age-matched TD in three treadmill walking conditions, e.g., baseline (adjusted to individual comfortable walking speed), variable speed (VS), or restricted foot width (RW). Then, we grouped similar muscle synergies using k-mean clustering and discriminant analyses from all datasets of individual synergy structures. Proportion tests exhibited six clusters of muscle synergies predominantly arising from children with CP and four clusters from children with TD. Also, the proportion of muscle synergies was significantly different in four of the CP-preferred clusters across conditions. Specifically, the proportion of the baseline condition was significantly different from VS and RW conditions in one and two clusters, respectively. The proportion was significantly different between VS and RW conditions in four clusters. Cadence and step lengths differed across conditions but not groups which makes the group differences in proportion even more notable. In contrast, step width, while significantly lower in CP, did not differ across conditions. Our findings demonstrate that muscle synergies in children with CP are more sensitive to changes in the external walking environment than in typically developing children.

## Introduction

Children with cerebral palsy (CP) have muscle coordination difficulties during walking due to alterations in the motor cortex, spinal motor neurons, or projections between them (Brouwer and Ashby, 1991). To visualize coordination patterns of individuals with brain injury, muscle synergies have been analyzed using a machine learning algorithm (Cheung et al., 2012). This analytical approach has also been used for examining muscle coordination patterns of walking in children with CP (Steele et al., 2015; Kim et al., 2018, 2021). As the result, previous studies found that children with CP have their own muscle coordination patterns (i.e., muscle synergies) that were relatively irregular between strides during walking when compared with healthy controls (Kim et al., 2018, 2021).

Muscle synergies have been proposed as motor commands of the central nervous system (Ting et al., 2015). This notion is supported by evidence that fundamental muscle synergy structures (synergy weights or vectors) rarely vary despite external environmental changes in walking, such as different gait speeds, slopes, type (e.g., Nordic or beam walking), or bodyweight support levels (Ivanenko et al., 2004; Gonzalez-Vargas et al., 2015; Sawers et al., 2015; Buurke et al., 2016; Rozumalski et al., 2017; Boccia et al., 2018; Kibushi et al., 2018; Saito et al., 2018). Likewise, overground and treadmill walking exhibited similar muscle synergy structures (Oliveira et al., 2016; Mileti et al., 2020). However, this has not yet been studied in individuals with CP.

Compared to children with typical development (TD), muscle synergies in CP were fewer and more varied between gait cycles, indicating decreased complexity and stability of motor control (Steele et al., 2015; Kim et al., 2018). Moreover, when classifying synergy structure composition using clustering analysis, some clusters are only present in CP but not in TD (Kim et al., 2021). Identifying CP-preferred muscle synergies at the individual level may facilitate the prescription of more individually customized rehabilitation. Desirably, understanding unique muscle synergies that may emerge in those with CP in various walking environments could provide insights into which muscles should be targeted during the training for neurorehabilitation.

This study aims to identify whether external environmental changes in walking influence muscle synergies in children with CP. We hypothesized that children with CP will show altered synergy structures in walking as in prior studies (Kim et al., 2018, 2021) as well as in two perturbed walking conditions, one with varying speeds, and one in which the walking path is narrowed. However, we expected that within the CP and TD groups baseline muscle synergies would be preserved despite these perturbations as shown previously. To test these hypotheses, we classified similar muscle synergy structures among different walking conditions using clustering analysis. Finally, we observed whether there were group- or environmental-preferred walking synergies using the proportion *z*-test.

## Materials and methods

### Participants

Participants included eight children with unilateral CP (age, 15.9 ± 29 yr; body mass, 60.6 ± 17.2 kg; height, 164.6 ± 11.2 cm; 4 left dominant; 4 right dominant) and eight children with TD within the same age range (age, 14.7 ± 3.6 yr; body mass, 69.8 ± 17.5 kg; height, 167.8 ± 5.2 cm; 8 right dominant). Seven females were in each group. Six children with CP were classified as Gross Motor Function Classification System (GMFCS) level I and two as level II. Exclusion criteria were botulinum toxin injections within 4 months, orthopedic surgery to the legs, or seizure within 6 months. The institutional review board approved the study protocol (#13-CC-0110) and informed written consent and assent were obtained for all participants.

### Procedures

**FIGURE 1:**
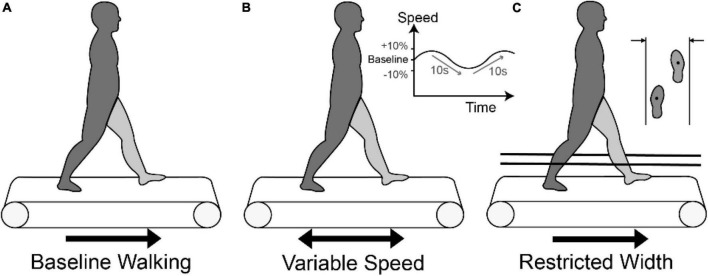
Three walking conditions: **(A)** Baseline walking (BW) on a treadmill at self-selected speed, **(B)** Variable speed walking (VS) condition with the treadmill speed sinusoidally moving between baseline and baseline ± 10% at a frequency of 0.1 Hz, and **(C)** Restricted width (RW) condition with strings placed at 46 cm.

Participants walked on the treadmill (Bertec TM-06-B, Columbus, OH, USA) while wearing a harness for safety at three different task conditions (Figure 1): baseline walking (BW) at self-selected speed without external intervention, variable speed (VS), and restricted width (RW). For BW, treadmill speed was initially estimated based on average pelvic velocity during overground walking. Each participant was provided ∼2 min to verify the comfortable speed. Then, for the VS condition, treadmill speed was continuously increased and decreased by 10% every 10 s from self-selected speed in a sinusoidal fashion. In the RW task, the width of the treadmill was constrained by placing two strings 46 cm apart parallel to both sides of the treadmill at mid-calf height. Initial pilot testing was performed in healthy participants to verify that the string did not interfere with walking, and then in participants with CP to determine a restricted distance which still allowed them to walk on the treadmill safely. We instructed participants to walk within the strings without contacting them if possible. Participants performed each task for 5 min with 5 min of rest between tasks.

### Biomechanical measurement

Muscle activation was recorded using a wireless electromyography (EMG) system (Trigno, Delsys, Boston, MA, USA). The EMG sampling rate was 1,000 Hz. Following SENIAM guidelines (Hermens et al., 1999), 16 surface electrodes were attached bilaterally to: tibialis anterior (TA), extensor hallucis longus (EH), lateral gastrocnemius (LG), soleus (SO), rectus femoris (RF), vastus lateralis (VL), semitendinosus (ST), and biceps femoris (BF) muscles. Placement of EMG electrodes was verified by manual muscle testing. Using ten motion capture cameras (Vicon, Lake Forest, CA), kinematic data were collected at 100 Hz and synchronized with the EMG system to detect gait events and measure gait parameters. All data were analyzed using Visual3D (C-Motion, Germantown, MD, USA).

To evaluate the effect of different conditions on walking we computed: number of gait cycles, dominant and non-dominant cadence (time between successive heel strikes reported in steps/min), dominant and non-dominant stride length excursion (sagittal plane foot excursion from toe-off heel strike during swing), and dominant and non-dominant stride width excursion (magnitude of frontal plane foot excursion during swing).

### Muscle synergy extraction

EMG data were high-pass filtered (3rd order Butterworth) at 35 Hz, full-wave rectified, and low-pass filtered (3rd order Butterworth) at 5 Hz. For muscle synergy extraction, EMG data were split into windows of 20 gait cycles, with 19 gait cycles of overlap between successive windows, supported by a previous study showing that reconstruction quality of muscle synergies was most stable when window size was 20 gait cycles (Oliveira et al., 2014). Here we focused on the relative contributions of each muscle within each window with EMG data normalized by the maximum activation value of each window. After splitting, EMG data sets concatenated with 20 cycles were time-interpolated to 2,000 points within each window. Therefore, a set of EMG data consisted of muscle × time matrices (EMGo) ranging from 0 to 1.

We used non-negative matrix factorization to extract muscle synergies from EMGo (Lee and Seung, 1999). This technique computed muscle synergies based on the following formula:


$$E\,M\,G_{0}=\sum_{i=1}^{n}{\text{W}}_{\text{i}}\,{\text{C}}_{\text{i}}+e,E\,M\,G_{r}=\sum_{i=1}^{n}{\text{W}}_{\text{i}}\,{\text{C}}_{\text{i}}$$

where *n* is the number of muscle synergies from 1 to 16, *i* is an identification number of each muscle synergy, W is a synergy structure (muscle × *n*) indicating weighting values of individual muscles for each synergy, C is a synergy activation (*n* × time) indicating time-varying synergy activation profiles, and *e* is residual error. EMGr is a reconstructed EMG matrix (muscle × time) resulting from the multiplication of W and C. To determine a valid number of muscle synergies, we calculated the variability accounted for (VAF) as follows:


V⁢A⁢F=1-(E⁢M⁢Go-E⁢M⁢Gr)2/E⁢M⁢Go2

VAF threshold was set at 90% (minimal n that VAF > 90%) in line with previous studies (Bowden et al., 2010; Clark et al., 2010; Dominici et al., 2011; Steele et al., 2015).

### Classifying similar muscle synergies

To classify similar muscle synergy structures among conditions, we used k-means clustering and discriminant analyses. The analytical procedure described in detail previously (Kim et al., 2021) is summarized here. The data matrix for clustering was 16 muscles × nW, where nW is the number of W matrices for all subjects (number of time windows × tasks × subjects × synergy numbers). For clustering, we used an iterative process to find the minimal number of clusters (k) identified by the intra-class correlation coefficient (ICC). The *k* value was set initially at the minimum number of synergies extracted from the 20 stride windows across the entire data set. Then, we used discriminant analysis to revise the cluster assignment if necessary. This supervised learning process aimed to optimize the linear or non-linear separation between clusters by projecting data into a subspace that maximized the variance between means of projected clusters and minimized the within cluster variance (Dixon and Brereton, 2009). Equality of cluster covariance matrices was assessed using Box’s *M* test (Box, 1949) and if equal, linear discriminant analysis (LDA) was used. Otherwise, quadratic discriminant analysis (QDA) was used (Dixon and Brereton, 2009). We then examined the requirement that synergy structures extracted from the same time window should be assigned into different clusters. If this condition was not satisfied, clustering was repeated with a sequential increase in k. Next, ICC examined the similarity of the synergy structures assigned to each cluster by discriminant analysis. To ensure an accurate *k* value, this process was repeated 1,000 times. Then, we selected the case showing the most frequent *k* value for the repetition and the highest mean ICC value across individual clusters.

To determine whether a cluster was preferred to a group, we calculated the proportion of synergies deriving from the TD or CP groups for each cluster using the two-proportion *z*-test (Sheskin et al., 2003). If the *z*-value was greater than 1.96 or lower than −1.96, the cluster was defined as CP-preferred or TD-preferred (*p* < 0.05), respectively. If the *z*-value was between −1.96 and 1.96, it was defined as a non-preferred cluster. Cluster ID was assigned according to the *z*-value rank. For example, C1, a CP-preferred cluster with the highest *z*-value, was the most frequently observed muscle synergy in CP.

### Statistical analysis

In each cluster, the proportion of synergies for individual walking conditions indicated whether the external environment changed muscle synergies for walking. Since the number of extracted synergies differed across conditions, each was normalized by the number of gait cycles (number of synergies/number of gait cycles × 100) in each condition. For instance, if 150 muscle synergies extracted from 200 gait cycles were assigned to a cluster, it was normalized as 75%. To compare proportions among walking conditions within a cluster, we conducted the χ test considering multiple comparisons for proportions (*p* < 0.05) then the two-proportion *z*-test for the *post-hoc* test with Bonferroni correction such that *p* < 0.0167 (0.05/3). Two-way mixed analysis of variance (2 groups × 3 conditions) was conducted to compare gait parameters (number of gait cycles, cadence, stride length excursion, stride width excursion) and mean number of synergies. Also, we used two-way mixed analysis of variance followed by Scheffe’s test to compare muscle synergy structures between clusters (Chvatal and Ting, 2012). Statistical analyses were performed using R (ver. 3.6.2) and SPSS (ver. 26) with significance set at *p* < 0.05.

## Results

As shown in Table 1, the mean number of synergies extracted from EMGo data set was significantly higher in the TD group compared to CP but were not significantly different among walking conditions.

**TABLE 1:** The number of muscle synerges among walking conditions and between groups.

Group	CP			TD			Between-condition comparison (*F*, *p*, *pη* ^2^, observed power)	Between-group comparison (*F*, *p*, *pη* ^2^, observed power)	Interaction (*F*, *p*, *pη* ^2^, observed power)
		
Condition	BW	VS	RW	BW	VS	RW			
The number of muscle synergeis	3.45 ± 0.47	3.42 ± 0.49	3.47 ± 0.49	3.99 ± 0.03	3.93 ± 0.12	3.97 ± 0.05	1.961, 0.160, 0.123, 0.271	9.393, 0.008, 0.402, 0.813	0.322, 0.728, 0.022, 0.096
95% confidence interval	3.05–3.84	3.00–3.83	3.07–3.88	3.97–4.01	3.83–4.02	3.94–4.01			

**FIGURE 2:**
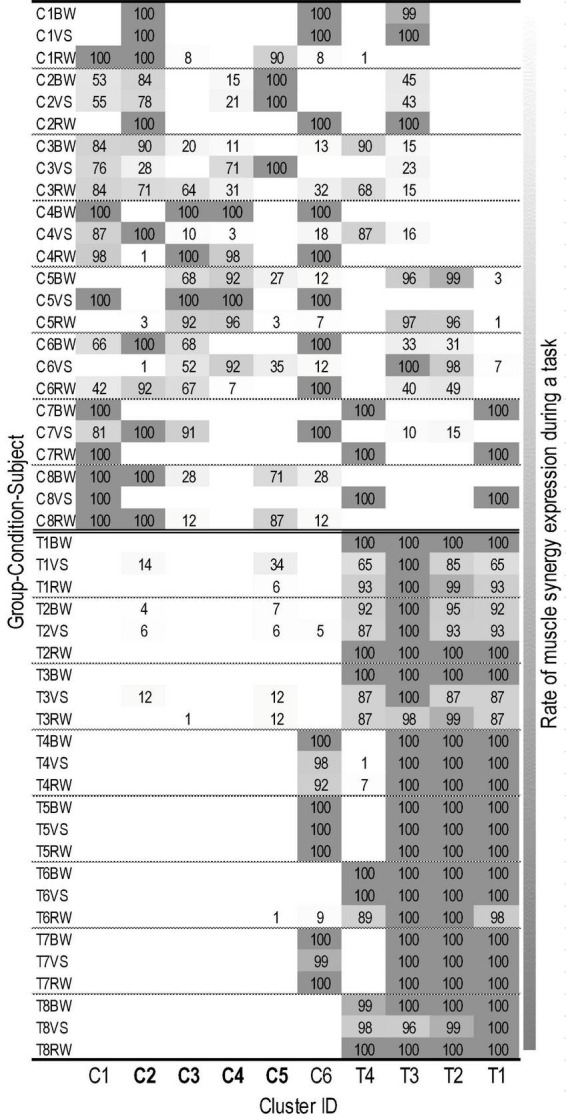
Individual assignment states of muscle synergies in 10 clusters. Labels on the Y-axis are combined by group (C, cerebral palsy; T, typical development), subject ID (1∼8), and walking condition (BW, baseline walking; VS, variable speed walking; RW, restricted width walking). The X-axis is labeled as cluster ID. The cluster where the proportion of muscle synergies differed significantly depending on the walking condition is presented in bold. The number of each cell represents the rate (%) of muscle synergy expression during a given walking task (i.e., 100 indicates that the muscle synergies corresponding to a cluster are extracted from all gait cycles during a task). Lower brightness in each cell indicates a greater expression rate of muscle synergies assigned in a cluster (refer to right column).

The iterative clustering algorithm found 10 distinct clusters across subjects and walking conditions (Figure 2). Based on *z*-values of the two-proportion test, we identified six CP-preferred (C1-6) and four TD-preferred muscle synergies (T1-4). We identified no non-preferred clusters according to z-scores, yet one CP-preferred cluster (C6, Figure 2) consistently contained synergies from three children with TD.

**FIGURE 3:**
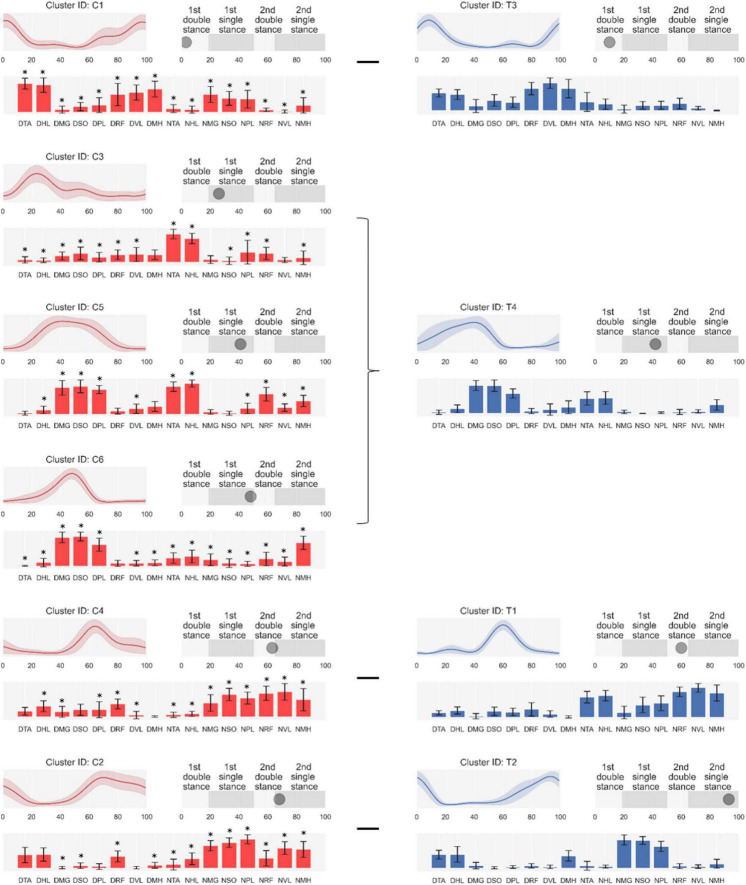
Synergy activations (top left), structures (low), and peak activation time in the gait cycle (top right). Cluster-ID is displayed above each subfigure (C, cerebral palsy; T, typical development). The left column consists of clusters specialized for cerebral palsy, and the right one is for typical development. Subfigures are arranged based on peak activation time from top to bottom for each column. Data are expressed as mean and standard deviation. The labels of bar plots are combined with leg side and muscle names as follows: D, dominant leg; N, non-dominant leg; TA, tibialis anterior; EH, extensor hallucis longus; LG, lateral gastrocnemius; SO, soleus; RF, rectus femoris; VL, vastus lateralis; ST, semitendinosus; BF, biceps femoris. *: Significant difference compared with the same muscle in children with typical development shown on the right of the figure (Scheffe’s test, *p* < 0.05).

As shown in Figure 3, we arranged clusters active in similar gait phases (i.e., first and second one-leg stance and double-leg stance) and compared muscle synergy structures. Scheffe’s test showed that group-preferred clusters have different muscle synergy structures despite activating at similar gait phases.

**FIGURE 4:**
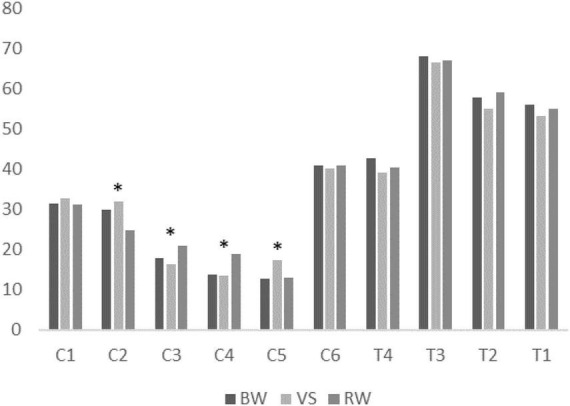
Mean proportion of muscle synergies for each walking condition in individual clusters. BW, baseline walking condition; VS, varying speed condition; RW, restricted width walking condition. *: Statistically significant difference among task conditions.

The proportion of muscle synergies among walking conditions was mainly and only significantly different in CP- preferred clusters. The χ test showed that the proportion of muscle synergies differed significantly depending on the walking condition within four of six CP-preferred clusters (C2, C3, C4, C5). For TD-preferred clusters, the proportion was not significantly different among walking conditions. *Post-hoc* tests showed that muscle synergies for the VS condition were significantly different in proportions of four clusters (C2, C3, C4, C5) compared to those for the RW condition. Muscle synergies during BW were significantly different in the proportion of C5 compared to the VS condition. Muscle synergies in BW condition were also significantly different from the RW condition in the proportion of C2 and C4. All proportion test results are presented in Table 2. Figure 4 shows the mean proportion of muscle synergies for each walking condition in individual clusters. Similarity among assigned synergies within a cluster, examined by ICC, ranged from 0.640 to 0.870 (mean: 0.759, standard deviation: 0.082).

**TABLE 2:** The results of proportion tests among walking conditions in each cluster.

ID	*X* test^(1)^		*Post-hoc* ^(2)^					
			BW vs. VS		BW vs. RW		VS vs. RW	
	*X*	*P*	*Z*	*P*	*Z*	*P*	*Z*	*P*
C1	1.202	0.548						
C2	18.469	**< 0.001**	–1.325	0.185	2.929	**0.003**	4.240	**< 0.001**
C3	9.606	**0.008**	0.842	0.400	–2.137	0.033	–2.967	**0.003**
C4	19.992	**< 0.001**	–0.050	0.960	–3.801	**< 0.001**	–3.736	**< 0.001**
C5	16.284	**< 0.001**	–3.505	**< 0.001**	–0.193	0.847	3.315	**0.001**
C6	0.014	0.993						
T4	2.065	0.356						
T3	0.140	0.932						
T2	1.870	0.393						
T1	0.641	0.726						

^(1)^Statistical significance: *p* < 0.05. ^(2)^Adjusted statistical significance: *p* < 0.0167 (marked as bold).

The number of analyzed gait cycles was not significantly different between groups [CP: 204 ± 44 (95% CI: 186–223), TD: 199 ± 36 (95% CI: 184–214); *F* = 0.231, *p* = 0.633] or among walking conditions [BW: 197 ± 43 (95% CI: 174–220), VS: 217 ± 45 (95% CI: 193–241), RW: 192 ± 28 (95% CI: 177–206); *F* = 1.754, *p* = 0.185]. Cadence and stride length excursion showed differences among walking conditions in contrast to stride width excursion which was the only parameter to show significant differences between groups (Table 3). No significant interactions between walking condition and group were found for any of gait parameters, even though interestingly, those with CP tended to decrease stride width excursion in the RW condition and those with TD tended to increase it compared to BW and VS. Still, dominant stride width excursion for TD remained significantly lower across all conditions than respective values in CP.

**TABLE 3:** Gait parameters among walking conditions and between groups.

Group	CP			TD			Between-condition comparison (*F*, *p*, *pη* ^2^, observed power)	Between-group comparison (*F*, *p*, *pη* ^2^, observed power)	Interaction (*F*, *p*, *pη* ^2^, observed power)
		
Condition	BW	VS	RW	BW	VS	RW			
Dominant cadence (stride/min)	51.21 ± 5.18	52.74 ± 5.20	52.05 ± 5.52	52.47 ± 2.92	54.08 ± 3.50	52.82 ± 3.27	25.602, **<0.001**, 0.646, 1.000	0.265, 0.615, 0.019, 0.077	0.979, 0.361, 0.065, 0.170
95% confidence interval	46.88–55.54	48.40–57.09	47.43–56.67	50.03–54.91	51.16–57.01	50.08–55.55			
Non-dominant cadence (stride/min)	51.20 ± 5.18	52.77 ± 5.24	2.06 ± 5.52	52.47 ± 2.92	54.08 ± 3.50	52.81 ± 3.27	26.482, **<0.001**, 0.654, 1.000	0.259, 0.618, 0.018, 0.076	0.977, 0.362, 0.065, 0.170
95% confidence interval	46.87–55.53	48.39–57.15	47.44–56.67	50.04–54.91	51.15–57.01	50.08–55.54			
Dominant stride length excursion (m)	0.587 ± 0.028	0.586 ± 0.032	0.572 ± 0.034	0.611 ± 0.090	0.616 ± 0.094	0.606 ± 0.091	6.828, **0.008**, 0.328, 0.824	0.759, 0.398, 0.051, 0.128	1.136, 0.327, 0.075, 0.205
95% confidence interval	0.563–0.610	0.559–0.612	0.543–0.600	0.535–0.686	0.537–0.695	0.530–0.682			
Non-dominant stride length excursion (m)	0.581 ± 0.022	0.582 ± 0.027	0.571 ± 0.029	0.608 ± 0.087	0.613 ± 0.091	0.605 ± 0.086	7.076, **0.003**, 0.336, 0.901	0.898, 0.359, 0.060, 0.143	1.147, 0.332, 0.076, 0.232
95% confidence interval	0.563–0.600	0.559–0.605	0.547–0.595	0.535–0.681	0.537–0.690	0.534–0.677			
Dominant stride width excursion (m)	0.054 ± 0.012	0.055 ± 0.011	0.048 ± 0.013	0.039 ± 0.005	0.039 ± 0.008	0.041 ± 0.016	0.691, 0.432, 0.047, 0.125	5.956, **0.029**, 0.298, 0.622	2.723, 0.117, 0.163, 0.355
95% confidence interval	0.044–0.064	0.045–0.064	0.037–0.059	0.034–0.043	0.032–0.046	0.028–0.055			
Non-dominant stride width excursion (m)	0.052 ± 0.015	0.054 ± 0.014	0.047 ± 0.013	0.037 ± 0.008	0.037 ± 0.007	0.041 ± 0.021	0.165, 0.724, 0.012, 0.068	4.457, 0.053, 0.241, 0.502	2.191, 0.157, 0.135, 0.301
95% confidence interval	0.039–0.065	0.043–0.066	0.037–0.058	0.030–0.044	0.031–0.044	0.024–0.059			

BW, baseline walking; VS, varying speed; RW, restricted width walking; CP, cerebral palsy; TD, typical development.

## Discussion

The purpose of this study was to determine if external walking manipulations influence muscle synergies in children with CP. As hypothesized, our analysis combining clustering algorithms and discriminant analysis found six CP-preferred and four TD-preferred muscle synergies during three walking conditions (BW, VS, and RW). However, the results did not support the hypothesis that muscle synergies in children with CP and TD would be preserved despite external walking environmental changes. Comparing the proportion of muscle synergies for the three walking conditions in each cluster, walking environment altered the structure of muscle synergies notably only in children with CP.

A previous study suggested that human neural networks use basic invariant factors for muscle patterns, e.g., muscle synergies, despite different walking speeds (Ivanenko et al., 2004). Our results suggest this is true in TD but not in children with CP. Importantly, only one child with CP here tended to use invariant muscle synergies for walking between BS and VS conditions. The rest with CP utilized different synergy patterns when the treadmill speed was varied (Figure 2). Interestingly, cadence and stride length excursion values were not different across groups, making the differences in synergies across conditions in CP even more notable.

The RW condition also showed a different proportion from BW in CP-preferred clusters (C2 and C4). Narrowing step width during walking challenges standing balance in the frontal plane (Arvin et al., 2016). A previous study demonstrated that muscle activations differed from baseline when compared with beam walking (Martino et al., 2015), but muscle synergies were generally similar between walking conditions in healthy subjects. These results support that the muscle synergy preferred by children with TD does not change with the environment as shown in our study. However, we found different proportions for RW in CP-preferred clusters (C2 and C4). This inconsistency may indicate that muscle synergies in children with CP change with the external environment.

We propose that external environmental changes could modify muscle coordination patterns more in children with CP than those with TD suggesting that children with CP are more reactive to external perturbations (Bulea et al., 2017). The human motor system may modulate muscle activation patterns in response to changes in the external environment (McGowan et al., 2010; Martino et al., 2015) depending on the kinematic and kinetic demands of walking conditions (Ivanenko et al., 2004). However, considering that children with TD showed invariant muscle synergies among conditions, we presume that a normally developed central nervous system might more effectively control descending and ascending neural signals to stabilize muscle coordination patterns from external environmental changes. Nevertheless, the expression “invariance of the muscle synergies” may lead to the misunderstanding that muscle coordination patterns never change among different walking conditions. Hence, it would be more appropriate for the injured human neural system such as CP to use flexible strategies and selectively utilize muscle coordination patterns according to the external environment.

In the gait parameters, the VS condition showed the highest cadence and stride length values when compared with other conditions, indicating that participants walked faster with larger stride length excursions during the VS condition. In RW, children with CP present a greater stride width excursion during swing than those of TD. Previous studies demonstrated a wider step width in CP compared to TD (Chang et al., 2011; Kurz et al., 2012), as shown here. The mean gait parameter values demonstrate that RW reduced stride width in children with CP while the TD group had a mean stride width less than the restricted value so had no need to alter this parameter. Surprisingly, the mean stride width excursion of the TD cohort increased slightly in the RW condition. Beam walking was more successful in narrowing step width in TD (Sawers et al., 2015); however, this walking condition would likely be too difficult if not hazardous for many with CP, so we decided to constrain width in a safer manner which in retrospect was not significantly constraining in TD, thus a limitation of our study design.

Considering that children with CP face various walking environments in daily life, it is important to identify whether and how muscle synergies in children with CP may adapt or change in different more challenging environments. In most previous studies, muscle synergies in children with cerebral palsy have been studied only during level walking at a self-selected walking speed (Steele et al., 2015; Kim et al., 2018, 2021). This study differs in that it examines changes in the muscle synergies of children with CP compared to a comparison group without CP in various external walking environments.

The small number of participants here likely limited the range of muscle synergies in given walking conditions. Hence, it would be beneficial to acquire larger datasets across multiple sites to better explore diverse muscle synergies in individuals with typical development and brain injury. Another limitation is that our findings are from a single session and cannot be generalized to long-term adaptive walking environments. Since long-term training could alter muscle synergies (Sawers et al., 2015), an individual’s motor experience should be considered when characterizing muscle synergies preferred to an external environment.

In conclusion, we found that changes in the external walking environment can modify the repertoire of muscle synergies in children with CP. In particular, CP-preferred synergies were vulnerable to external environmental changes. Our analytical technique provides a quantitative approach to differentiate muscle synergies among various walking conditions at an individual level. Also, our approach would be helpful to identify whether individually tailored gait rehabilitation focused on walking perturbations improves muscle coordination patterns.

## Data availability statement

The original contributions presented in this study are included in the article/supplementary material, further inquiries can be directed to the corresponding author.

## Ethics statement

The studies involving human participants were reviewed and approved by the Institutional Review Board of NIH #13-CC-0110. Written informed consent to participate in this study was provided by the participants’ legal guardian/next of kin.

## Author contributions

TB and YK performed experiments and analyzed data. YK prepared figures and drafted the manuscript. All authors conceived and designed the research, interpreted results of experiments, edited and revised the manuscript, and approved final version of manuscript.
